# Developmental Increase of Neocortical Presynaptic Efficacy *via* Maturation of Vesicle Replenishment

**DOI:** 10.3389/fnsyn.2019.00036

**Published:** 2020-01-15

**Authors:** Grit Bornschein, Simone Brachtendorf, Hartmut Schmidt

**Affiliations:** Carl-Ludwig-Institute for Physiology, Medical Faculty, University of Leipzig, Leipzig, Germany

**Keywords:** layer 5 pyramidal neurons, action potential trains, synaptic efficacy, failures, paired-pulse ratios, replenishment, vesicle pools, presynaptic calcium signals

## Abstract

The efficacy of neocortical synapses to transmit during bursts of action potentials (APs) increases during development but the underlying mechanisms are largely unclear. We investigated synaptic efficacy at synapses between layer 5 pyramidal neurons (L5PNs) during development, using paired recordings, presynaptic two-photon Ca^2+^ imaging, and numerical simulations. Our data confirm a developmental increase in paired-pulse ratios (PPRs). Independent of age, Ca^2+^ imaging revealed no AP invasion failures and linear summation of presynaptic Ca^2+^ transients, making differences in Ca^2+^ signaling an unlikely reason for developmental changes in PPR. Cumulative excitatory postsynaptic current (EPSC) amplitudes indicate that neither the size of the readily-releasable pool (RRP) nor replenishment rates were different between age groups, while the time-courses of depression differed significantly. At young synapses, EPSCs depressed rapidly to near steady-state during the first four APs, and synaptic failures (F_syn_) increased from 0 to 30%. At mature synapses this drop was significantly slower and strongly biphasic, such that near steady-state depression was reached not before 18 APs with F_syn_ remaining between 0 and 5%. While young synapses reliably transmitted during pairs of APs, albeit with strong depression, mature synapses maintained near 100% transfer efficacy with significantly less depression during high-frequency bursts of APs. Our analysis indicates that at mature synapses a replenishment pool (RepP) is responsible for their high efficacy during bursting activity, while this RepP is functionally immature at young synapses. Hence, our data provide evidence that the functional maturation of a RepP underlies increasing synaptic efficacy during the development of an excitatory cortical synapse.

## Introduction

Synaptic efficacy is the capacity of a presynaptic input to influence the postsynaptic neuron (López, 2002). A synapse can be considered as a device that receives trains of presynaptic action potentials (APs) and affects the postsynaptic output through graded analog responses (London et al., 2002). In this process, synapses do not transmit each AP identically but do so in a manner that is dependent on the history of activity of the synapse. During trains of APs, synaptic transmission may either show short term depression (STD) or short term facilitation (STF), sometimes referred to as “phasic” or “tonic” synapses, respectively (Pan and Zucker, 2009; Neher and Brose, 2018). Hence, to evaluate changes in synaptic efficacy it is required to study also more complex synaptic signals in addition to single excitatory postsynaptic currents (EPSCs) and paired pulses (Markram and Tsodyks, 1996).

On the presynaptic site, transmission efficacy depends on the number of release sites (*N*) occupied by release-ready synaptic vesicles (SVs; *N*_occ_ in the following) and their average vesicular release probability (*p*_v_; Quastel, 1997). The SVs occupying the *N*_occ_ can be released by an AP and are referred to as the “readily releasable pool” (RRP) here (Rizzoli and Betz, 2005). During a train of APs, SVs from the RRP are progressively used and sustained information transfer efficacy now depends on the speed of replenishment of SVs into the RRP, i.e., the restoration of *N*_occ_, and their *p*_v_, which may increase. As a consequence, either synaptic depression or synaptic facilitation result (Quastel, 1997; Neher and Brose, 2018; Schmidt, 2019).

Synapses that show STD during high-frequency trains of APs include the calyx of Held and cerebellar mossy fiber terminals. The RRP of these synapses has been suggested to be subdivided into two pools, a “fast releasable pool” and a “slow releasable pool”. The main mechanism of STD during ongoing activation of these synapses is thought to be the progressive depletion of the fast releasable SVs (Sakaba, 2006; Wölfel et al., 2007; Hallermann et al., 2010; Ritzau-Jost et al., 2018).

Among the synapses showing STF during high-frequency trains of APs are cerebellar parallel fiber synapses. Recent studies suggest that these synapses operate with sequential pools of SVs and harbor a replenishment pool (RepP) in series with the RRP. During a train of APs, a very rapid and reversible transition of SVs from RepP to RRP temporarily increases *N*_occ_ and forms the basis of their lasting high-frequency facilitation (Valera et al., 2012; Brachtendorf et al., 2015; Miki et al., 2016; Doussau et al., 2017).

Glutamatergic synapses in the young neocortex show strong STD during high-frequency bursts of APs. STD becomes attenuated and may eventually even convert to moderate STF during postnatal development (Feldmeyer and Radnikow, 2009), with some layer specificity (Lefort and Petersen, 2017). The molecular mechanisms underlying this developmental increase in synaptic efficacy during bursts are largely unclear. In the traditional interpretation developmental alterations in *p*_v_ lead to altered short-term plasticity (STP, Zucker and Regehr, 2002) However, we recently found at glutamatergic synapses between layer 5 pyramidal neurons (L5PNs) that neither *p*_v_ (~0.63) nor *N* (~8) change during development of these synapses (Bornschein et al., 2019), thus, posing a problem for the traditional interpretation of mechanisms underlying changes in STP. In light of the published results from synapses displaying either STD or STF (reviewed in Neher and Brose, 2018; Schmidt, 2019), we hypothesize that differences in the organization of SV pools and/or in the speed of replenishment of the RRP during trains of APs could be responsible for the developmental increase in synaptic efficacy at glutamatergic neocortical synapses.

To test this hypothesis, we studied synaptic transmission at synapses formed between pairs of L5PNs in the young and mature cortex during high-frequency trains of APs. We found that release from young synapses depressed significantly faster than release from mature synapses, while relative EPSC amplitudes finally dropped to comparable steady-state levels. Single bouton two-photon Ca^2+^ imaging revealed linear summation of AP-mediated Ca^2+^ signals in both age groups, indicating that depression of Ca^2+^ influx is not involved. By analyzing cumulative EPSC amplitudes and time-courses of depression we extract information about RRP replenishment and the organization of SV pools. While replenishment rates and the initial size of the RRP were similar between age groups, our results indicate the presence of a RepP in mature synapses that has not fully developed at young synapses and that constitutes the increased train-transmission fidelity of mature synapses.

## Materials and Methods

### Slice Preparation and Electrophysiology

P8–10 and P21–24 C57BL/6J mice of either sex were decapitated under deep isoflurane (Curamed) inhalation anesthesia. The brain was excised rapidly and placed in cooled (0–4°C) artificial cerebrospinal fluid (ACSF) containing (in mM): 125 NaCl, 2.5 KCl, 1.25 NaH_2_PO_4_, 26 NaHCO_3_, 1 MgCl_2_, 2 CaCl_2_, and 20 glucose, equilibrated with 95% O_2_ and 5% CO_2_ (pH 7.3–7.4). Coronar slices (250–300 μm thick) were cut from the S1 region with a HM 650 V vibratome (Microm), incubated for 30 min at 35°C, and subsequently stored at room temperature (22°C). For experiments slices were transferred to a recording chamber and continuously perfused with 2–3 ml ACSF/min supplemented with 10 μM (-)-bicucculine methiodide (Tocris) at 30–32°C. Unless stated otherwise, chemicals were from Sigma–Aldrich.

Patch pipettes were prepared from borosilicate glass (Hilgenberg) with a PC-10 puller (Narishige) and had final resistances of 6–8 MΩ. The standard pipette solution contained (in mM): 150 K-gluconate, 10 NaCl, 3 Mg-ATP, 0.3 Na-GTP, 0.05 EGTA, 10 HEPES, dissolved in purified water. The pH was adjusted to 7.3 with KOH.

Patch-clamp recordings from pairs of L5PNs were performed under optical control (BX51WI, Olympus), using an EPC10/2 amplifier and Patchmaster software (version v2x73.2, HEKA). EPSCs were recorded in the whole-cell configuration at a holding potential (V_hold_) of −80 mV (corrected for liquid junction potential of 16 mV), filtered at 5 kHz and sampled at 20 kHz. Holding current (I_hold_) and series resistance (R_s_) were monitored continuously. R_s_ values were compensated continuously to a value between 10–15 MΩ. Experiments were rejected if the uncompensated R_s_ exceeded 30 MΩ or if I_hold_ exceeded −500 pA. Presynaptic cells were stimulated in on-cell configuration (100–500 pA, 1–2 ms) at inter-series intervals ≥10 s.

### M-V Analysis

Mean-variance (M-V) analysis was performed as described previously (Bornschein et al., 2019) assuming binominal release statistics (Clements and Silver, 2000). EPSC amplitudes were recorded at different [Ca^2+^]_e_ of 0.5, 1, 2, 5, 10 mM with [Mg^2+^]_e_ being adjusted, respectively, to 2.5, 2, 1, 0, 0 mM (≥50 repetitions per concentration). Variances (*σ^2^*) of first and second EPSCs (Scheuss et al., 2002), were plotted against the corresponding mean amplitudes (*I*) and data of the first EPSCs fitted by a parabola:

$$\sigma^{2}=Iq-\frac{I^{2}}{N}(1+CV_{\text{II}}^{2})+qICV_{\text{I}}^{2}$$

where *q* is the quantal size, *N* a binominal parameter, and *CV*_I_ and *CV*_II_ the coefficients of intrasite and intersite quantal variability, assumed to be 0.3 (Clements and Silver, 2000). The variance of the variance was calculated (according to Meyer et al., 2001).

### Fluorescence Imaging

Ca^2+^ imaging experiments were performed at presumed presynaptic terminals of axon collaterals as described previously (Schmidt et al., 2013; Kusch et al., 2018; Bornschein et al., 2019). Briefly, L5PNs were equilibrated with EGTA-free, Fluo-5F (200 μM, Invitrogen) and Alexa-594 (50 μM, Invitrogen) containing pipette solution *via* somatic whole-cell patch-pipettes. Fluorescence transients were elicited by somatically induced APs (2–4 nA stimulus for 1–2 ms) and recorded in linescans at 300–500 Hz temporal resolution with a custom-build two-photon microscope based on a Fluoview-300 system (Olympus), using a 60×/0.9 N.A. objective, a mode-locked Ti:sapphire laser (Tsunami, Newport-Spectra Physics, set to a center wavelength of 810 nm), and a Pockels cell (350–80 KD*P, Conoptics). Each bouton was recorded 3–5 times. Typically at least four boutons per L5PN were recorded and averages per cell were calculated. The volume-averaged fluorescence signals were filtered (HC647/75, Semrock HC525/50, 720-SP, AHF), detected by two external PMT modules (H7422-40, Hamamatsu; PMT-02M/PMM-03, NPI electronics; monitoring epi- and trans-fluorescence, respectively at fixed PMT voltages), and digitized with the Fluoview system. The Ca^2+^-dependent green fluorescence was normalized to the Ca^2+^-insensitive red fluorescence and expressed as background-corrected ΔG/R (Sabatini et al., 2002).

### Models of Ca^2+^ Dynamics and Transmitter Release

Models for Ca^2+^-dependent SV fusion and replenishment were transformed into the corresponding ordinary differential equations and numerically solved using Mathematica 12.0 (Wolfram Research) as described previously (Doussau et al., 2017; Bornschein et al., 2019). Release triggering Ca^2+^ signals were simulated as repeated triple-exponential functions, spaced by the interstimulus intervals (ISI), and adjusted to match amplitude and time-course of the estimated AP-mediated Ca^2+^ signal at the release sensor; the resting Ca^2+^ was 38 nM (Bornschein et al., 2019). The Ca^2+^ signals were fed into the allosteric five-site release sensor model (Lou et al., 2005) that was modified to represent synaptotagmin-1 by scaling the off-rate of the first Ca^2+^-binding reaction by 0.5 (Schmidt et al., 2013). The *p*_v1_ was calculated by integrating over all fused states and was 0.63 under these conditions. The release sensor model was supplemented by the RepP and/or an infinite reserve pool. The models were fit to the experimental data by setting the size of the RepP to a given value and subsequently manually adjusting the replenishment rates k_0_ or k_1_ and k_2_ until the root mean square deviation between data and simulation reached a minimum.

### Analysis and Statistics

Data were analyzed using custom written routines in Igor Pro 7 (Wavemetrics). Spontaneous EPSC (sEPSCs) were analyzed with the Neuromatic procedures for Igor^1^. The detection threshold was set to −2.5 pA. Amplitudes larger than −15 or −20 pA were accepted as sEPSCs for young and mature pairs, respectively. All summarized data are shown as median and IQRs irrespective of their statistical distribution; in box plots mean values are included as dashed lines. Normality was tested using Shapiro–Wilk test. Normally distributed data were compared with the *t*-test (two groups); non-normally distributed data were compared with the Mann–Whitney-*U* rank sum test (MWU; two groups). All statistical tests were two-tailed. *P*-values are indicated as *P ≤ 0.05, **P ≤ 0.01 and ***P ≤ 0.001. The number of experiments was chosen sufficiently high to achieve consistent results and permit statistical analysis when appropriate. Statistics were performed with Sigma Plot 11.0 (Dundas Software).

## Results

### PPR of EPSCs Increases During Development

**Figure 1:**
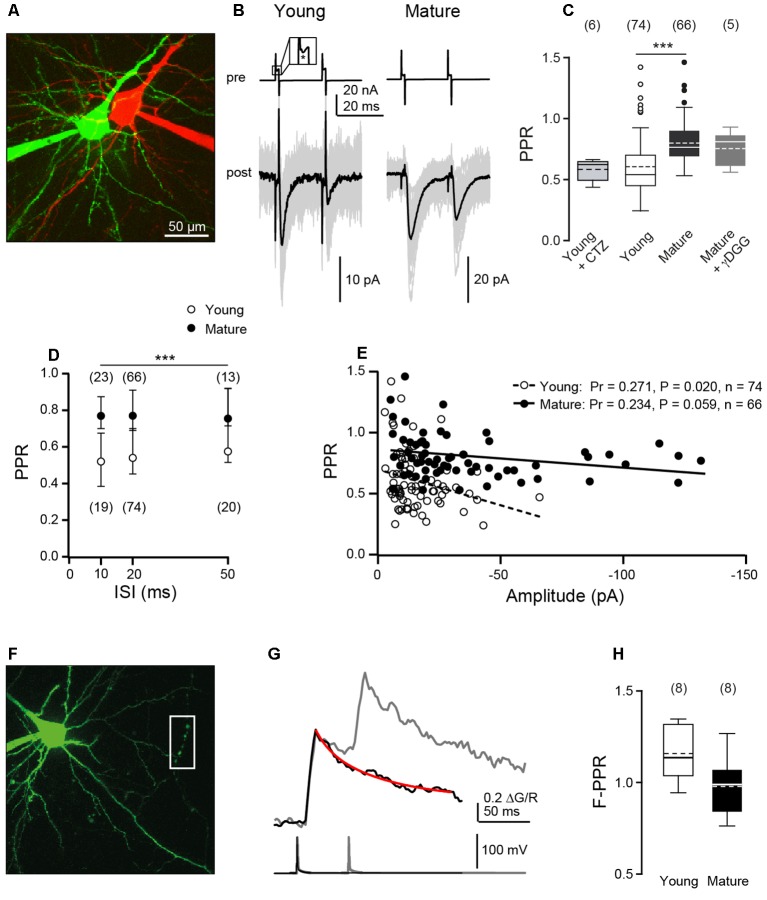
Paired-pulse ratios (PPRs) of excitatory postsynaptic currents (EPSCs) decrease during development while Ca^2+^ signals sum linearly. **(A)** Two-photon image of a pair of connected layer 5 pyramidal neurons (L5PNs) in S1 cortex in an acute slice from a P9 mouse. Pre- and post-synaptic cells were filled with red or green fluorescent dyes, respectively. **(B)** Presynaptic cells from a young (P9; left) and a mature mouse (P23, right) were stimulated with paired-pulses at interstimulus intervals (ISIs) of 20 ms [*top*; inset: action current (AC) marked by asterisk] and corresponding EPSCs (bottom; individual recordings in gray, *n* ≥ 20, average in black) were recorded from the postsynaptic neurons. **(C)** Summary of (PPRs = A2/A1) at 20 ms ISI in young (P8–10) and mature (P21–24) L5PNs. Note that application of γ-D-glutamylglycine (γDGG) or cyclothiazide (CTZ) did not significantly affect PPR. Box plots show median and IQR, mean as dashed line, whiskers indicate farthest point within 1.5-fold IQR, dots indicate outliers, numbers of cell pairs in brackets [***P < 0.001, Mann–Whitney-*U* (MWU) test]. **(D)** PPRs obtained for young and mature cell pairs at the indicated ISIs. PRRs in the mature were significantly larger than in the young (median ± IQR, *n* in brackets, ***P < 0.001, two-way ANOVA) while within a given age-group PPRs were independent of the range of ISIs tested. **(E)** Correlation between PPR and averaged first amplitude for individual cell pairs. Pr, Pearson’s correlation coefficient. **(F)** L5PN in a slice from a P9 mouse filled *via* a patch pipette with 200 μM Fluo-5F and 50 μM Alexa-594. Action potential (AP)-induced fluorescence changes were recorded in line scans from the presumed presynaptic boutons located on the axon collateral outlined by the white box. **(G)** Example of averaged ΔG/R signals from five boutons of one cell (top) elicited by one (black) or two APs (gray; ISI of 50 ms; bottom) superimposed. The red line represents an exponential fit to the single-AP response, which was used to calculate the second amplitude. **(H)** Median fluorescence PPRs (F-PPR) indicate a linear summation of AP-evoked Ca^2+^ signals in both age groups.

To analyze synaptic efficacy during repetitive activity, we performed paired patch-clamp recordings from connected L5PNs in acute brain slices of young (P8–10) and matured (P21–24) mice. Presynaptic cells were stimulated on-cell and postsynaptic EPSCs were recorded in the whole-cell configuration. For verification of cell-type, at the end of experiments also the patch of the presynaptic neuron was ruptured and both neurons were filled with fluorescent dyes and two-photon imaged (Figure 1A).

First, we performed paired-pulse experiments at ISIs of 20 ms and quantified paired-pulse ratios (PPRs) of EPSC amplitudes (A2/A1). We found paired-pulse depression in both age windows, albeit with significantly less depression at mature (median PPR = 0.77, 0.69–0.90) than at young synapses (PPR = 0.54, 0.45–0.70, *P* < 0.001, MWU test; Figures 1B,C), which is consistent with a previous observation at these synapses (Frick et al., 2007).

To test the site of depression, we used the low-affinity antagonist γ-D-glutamylglycine (γDGG), which relieves the effects of desensitization and saturation at AMPA receptors (Wadiche and Jahr, 2001; Crowley et al., 2007), at mature synapses. We found that application of γDGG (1–2 mM) clearly reduced EPSC amplitudes (A1 reduced to 36%, 17–39%, *n* = 5), while PPR remained unaffected (0.81, 0.61–0.86, *n* = 5, *P* = 0.822; Figure 1C) in comparison to control. The small-sized EPSCs at young synapses (~12 pA; see Bornschein et al., 2019) impeded the use of γDGG here. Since possible effects of desensitization are more pronounced at shorter than at larger ISIs (Chanda and Xu-Friedman, 2010) we analyzed PPR at ISIs of 10–50 ms in both age groups (Figure 1D). For all ISIs, PPR was smaller at young than at mature synapses (*P* < 0.001, two-way ANOVA). However, within an age group the PPR showed no significant dependence on the range of ISIs tested (mature: PPR_10 ms_ = 0.77, 0.70–0.88, *n* = 23; PPR_50 ms_ = 0.75, 0.71–0.94, *n* = 13; young: PPR_10 ms_ = 0.52, 0.38–0.68, *n* = 19; PPR_50 ms_ = 0.58, 0.51–0.92, *n* = 20; *P* = 0.210). This is consistent with our previous finding of non-detectable receptor saturation also at young L5PNs (Bornschein et al., 2019). To further investigate this point, we applied cyclothiazide (CTZ, 50 μM), which prevents AMPA receptors from desensitization (Chanda and Xu-Friedman, 2010) but also has different described presynaptic effects (Ishikawa and Takahashi, 2001). Application of CTZ neither had a significant effect on EPSC amplitudes (reduction of A1 to 78%, 70–80%, *P* = 0.31; *n* = 6) nor on the PPR recorded at young synapses at an ISI of 20 ms (0.63, 0.54–0.64; Figure 1C; *P* = 0.5, MWU test). Together these data indicate a predominantly presynaptic origin of synaptic depression in both age groups and argue against a significant contribution of postsynaptic receptor desensitization or saturation.

We continued by analyzing the relationship between PPRs and the first EPSC amplitudes. PPRs significantly decreased with increasing initial amplitude at young synapses (*P* = 0.020, Pr = 0.271, *n* = 74, Pearson product-moment correlation) but to a lesser and not significant extent at mature synapses (*P* = 0.059, Pr = 0.234, *n* = 66; Figure 1E). This may indicate that mature synapses can compensate more effectively for the consumption of SVs of the RRP than young synapses.

In the absence of receptor saturation and SV replenishment, the PPR is given by PPR = A2/A1 = *p*_v2_/*p*_v1_ * (1−*p*_v1_). Using *p*_v1_ of 0.63 (Bornschein et al., 2019), with the highest possible *p*_v2_ of 1 an upper limit for the PPR of 0.59 results. Experimental PPRs at young synapses are close to this limit, while PPRs at mature synapses clearly exceed it, indicating that RRP replenishment is a factor of PPR at these synapses.

In addition to replenishment, the number of release sites may reversibly increase within the ISI of paired-pulse experiments (Valera et al., 2012; Brachtendorf et al., 2015; Miki et al., 2016; Doussau et al., 2017). In multi-probability fluctuation analysis of paired-pulse experiments, this would be revealed by a deviation of the mean-variance data of second EPSC amplitudes from the parabola obtained for the corresponding first amplitudes (Clements and Silver, 2000). In our investigations, we found that A2 mean-variance data fall to the same parabola as A1 mean-variance data (Supplementary Figure S1; see Bornschein et al., 2019). Hence, there are no indications for changes in the number of releases sites during repeated synaptic activations.

In summary, this initial series of experiments confirms a developmental decrease in PPD (Frick et al., 2007), indicates that the origin of synaptic depression is presynaptic, and that replenishment of SVs is a factor of PPR, while activity-dependent changes in the number of release sites are not.

### Linear Summation of Presynaptic Ca^2+^ Signals

The initial *p*_v_ and *N*, as well as single AP-mediated presynaptic Ca^2+^ transients are not different between the two age groups investigated here (Bornschein et al., 2019). We now tested, whether depression of presynaptic Ca^2+^ signals may contribute to EPSC depression. Towards this end, L5PNs of both age groups were equilibrated with the green fluorescent Ca^2+^ indicator dye Fluo-5F (*K*_D_ ~1.3 μM; Bornschein et al., 2019) and red fluorescent Alexa-594. AP-mediated fluorescence signals were recorded from boutons located on recurrent axon collaterals and quantified as ΔG/R (Figure 1F). Pairs of APs were elicited at an ISI of 50 ms, whereupon every AP induced a Ca^2+^ transient in boutons of both age groups, i.e., we found no indications for AP-invasion failures (see Bornschein et al., 2019). The Ca^2+^ signals summed linearly in both age groups (Figures 1G,H) as indicated by the fluorescence PPRs (F-PPRs) of ΔG/R signals of ~1 (young: 1.14, 1.04–1.32, *n* = 8, *P* = 0.06, *t*-test vs. 1; mature: 0.99, 0.84–1.07, *n* = 8, *P* = 0.80). If at all, F-PPRs tended to be slightly but not significantly smaller in mature boutons than in young ones. Hence, differences in presynaptic Ca^2+^ signal summation appear not to account for the observed developmental increase in the PPR of EPSCs.

### Steady-State Rates of SV Replenishment Are Independent of Age

**Figure 2:**
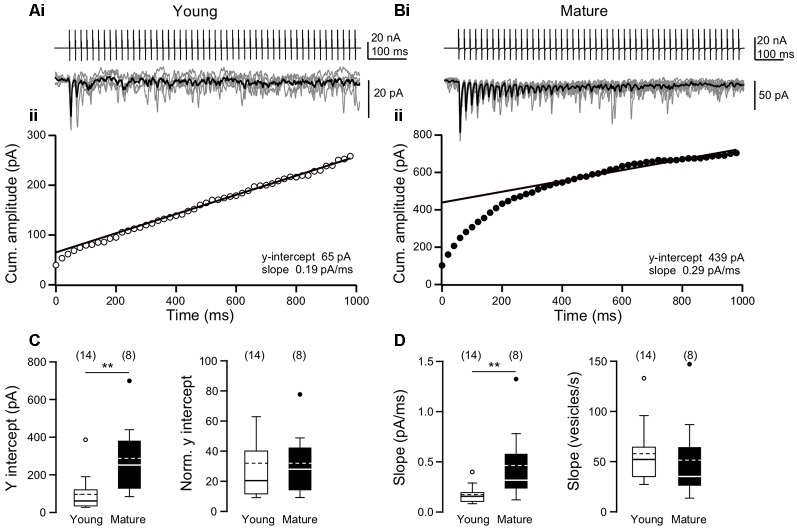
Stability of readily releasable pool (RRP) size and steady-state replenishment rates during development. **(Ai)** Example of 10 consecutive EPSC trains (gray; average in black; bottom), each induced by 50 ACs at an ISI of 20 ms (top) in the presynaptic neuron (P10). **(ii)** Cumulative EPSC amplitudes from the EPSCs in **(i)** plotted over time with a linear fit to the steady-state phase. **(B)** As in **(A)**, but for a pair of connected L5PNs from a P22 mouse. **(C)** Summary of y-intercepts (left) and y-intercepts normalized to quantal size (*q*) of 3 pA and 9 pA in young and mature L5PNs, respectively [right; cf. Bornschein et al., 2019; boxes: median ± IQR, dashed line: mean, whiskers: farthest point within 1.5-fold IQR, number of experiments in brackets; **P = 0.006, MWU test]. **(D)** Summary of slopes (left) and slopes normalized to quantal size (right; **P = 0.01, MWU test).

To directly test the idea of differential SV replenishment between age groups, we applied high-frequency trains of 50 APs and examined cumulative EPSC amplitudes (Schneggenburger et al., 1999). The decay of EPSCs was fast enough such that at an ISI of 20 ms no tonic component build up during the trains. At synapses of both age groups, EPSCs depressed to a steady-state level of ~10% of A1. Since *p*_v_ is large at L5PN synapses (>0.6; Bornschein et al., 2019) it is likely that SV replenishment is the limiting process during the steady-state phases and that the initial RRP has been used-up substantially. We calculated cumulative EPSC amplitudes during the AP trains, fitted lines to the steady-state phases of the curves, and extrapolated the fits to the y-axis intercept (Figures 2A,B). The y-axis intercepts of these line-fits relate to the size of the decrement of the RRP during the train and the slopes of the line-fits are a measure of the steady-state replenishment rate of SVs during the train (Schneggenburger et al., 1999; Neher, 2015). In absolute terms the y-intercepts were significantly smaller in young (61 pA, 35–122 pA, *n* = 14) than in mature synapses (252 pA, 122–410 pA, *n* = 8, *P* = 0.006, MWU test). However, the quantal size (*q*) is 3-fold smaller in young than in mature synapses (Bornschein et al., 2019). Considering this difference by normalizing the y-intercepts to the corresponding *q*-values of young (3 pA) and mature synapses (9 pA) yielded values that were no longer significantly different between age-groups (young: 20, 12–41; mature: 28, 14–46; *P* = 0.759, MWU test). Since *N* and *p*_v_ are developmentally stable (Bornschein et al., 2019), these findings suggest that the RRP did not change during synapse maturation.

In absolute values, we found also significantly higher slopes at mature (0.32 pA/ms, 0.21–0.68 pA/ms, *n* = 8) than at young synapses (0.16 pA/ms, 0.10–0.21 pA/ms, *n* = 14; *P* = 0.010, MWU test; Figure 2D). However, again considering the difference in *q* by normalization to the corresponding values we obtained similar replenishment rates for both age groups (young: 52 vesicles/s, 35–69 vesicles/s, *n* = 14; mature: 35 vesicles/s, 23–76 vesicles/s, *n* = 8; *P* = 0.322, MWU test). In summary, the cumulative EPSC analysis suggests that neither differences in the initial size of the RRP nor differences in the speed of its replenishment account for the developmental differences in STP.

### Age-Dependent Differences in Synaptic Efficacy During Bursting Activity

**Figure 3:**
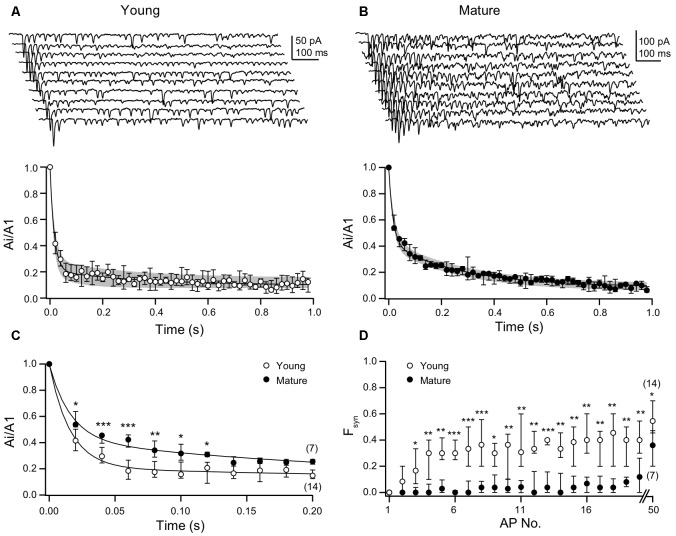
Young synapses have lower efficacy during bursting activity than mature synapses. **(A)** Top: examples of 10 consecutive EPSC trains each induced by 50 ACs at 20 ms ISIs in the presynaptic neuron of a P10 mouse. Bottom: median EPSC amplitudes and IQRs from young L5PN pairs (*n* = 14) normalized to first amplitudes (A1, double-exponential fit in black, IQR in gray). **(B)** As in **(A)**, but for mature L5PNs (*n* = 7). **(C)** Ai/A1 ratios are significantly higher in mature than in young L5PNs for the first seven EPSCs (2nd: *P = 0.012, 3rd+4th: ***P < 0.001, 5th: **P = 0.009, 6th: *P = 0.028, 7th: *P = 0.015; MWU test). **(D)** Synaptic failures (F_syn_) during the train occurred significantly earlier and more often in young than in mature connections following the third activation (*P < 0.05, **P < 0.01, ***P < 0.001; MWU test).

We proceeded by analyzing the decay of EPSC amplitudes during the high-frequency trains of APs in more detail (Figures 3A,B). EPSC amplitudes in the train were normalized to the first amplitude, i.e., they were expressed as Ai/A1. In both age groups, the time-courses of depression of the Ai/A1 ratios were best described by double-exponential fits, albeit with clear differences between young and mature synapses. While the amplitude of the fast component was larger in young (0.79, 0.71–0.81, *n* = 14) than in mature synapses (0.57, 0.54–0.62, *n* = 7), the amplitudes of the slow components showed a reciprocal relationship (young: 0.12, 0.11–0.14; mature: 0.33, 0.33–0.34). On the other hand, the time constants of the fits and the relative steady-state amplitudes were similar among age groups (young: τ_1_ = 16, 13–17 ms, τ_2_ = 246, 90–300 ms; y_0_ = 0.097, 0.076–0.156; mature: τ_1_ = 16, 16–21 ms, τ_2_ = 276, 270–290 ms; y_0_ = 0.089, 0.054–0.114). Thus, EPSC amplitudes rapidly dropped to near steady-state within four APs in young synapses (Figure 3A), whereas in mature synapses near steady-state depression was not reached before 18 APs (Figure 3B). The latter being due to the pronounced second component of the biphasic time-course of depression. Statistical comparison of Ai/A1 ratios yielded significantly higher values for the 2nd to 7th stimulation in mature synapses as compared to young synapses (Figure 3C; Supplementary Table S1).

In a subset of pairs with relatively large EPSC amplitudes, we could analyze the amplitudes of sEPSC following the high-frequency trains (Supplementary Figure S2). These sEPSCs are likely to originate in part from the L5PN synapse under investigation and to include miniature EPSCs. If receptor desensitization or saturation would contribute to the differences in the time-courses of depression during the trains, we should have been able to detect a fraction of smaller sEPSCs immediately after the train that subsequently recovers. However, we detected no such small EPSCs and found no correlation between sEPSC amplitudes and recording time. Together with our analysis of PPRs in the presence of γDGG or CTZ (Figure 1C), this is further evidence for a mainly presynaptic origin of depression in both age windows.

Synaptic failures (F_syn_) contributed to EPSC amplitudes during the trains. In young and mature synapses the initial *p*_v_ is similarly high, which, together with *N* of ~8 (Bornschein et al., 2019), resulted in an initial number of F_syn_ of ~0 in both age groups. Yet, already for the second AP F_syn_ increased from 0 in the young and raised to a steady-state level of ~30–40% during the first ~4 APs, which reflects the course of EPSC depression. In contrast, transmission at mature synapses remained highly reliable during the trains with F_syn_ remaining <5% for the first 18 APs (Figure 3D; Supplementary Table S2). Only thereafter F_syn_ increased but remained significantly (Supplementary Table S2) smaller than in the young even for the 50th AP. In the absence of age-dependent differences in the initial sizes of the RRPs and in the steady-state rates of their replenishment (Figure 2), these data suggest that at mature synapses a further process is operational that has not yet matured in the young.

### Organization of SV Pools at Young and Mature Synapses

**Figure 4:**
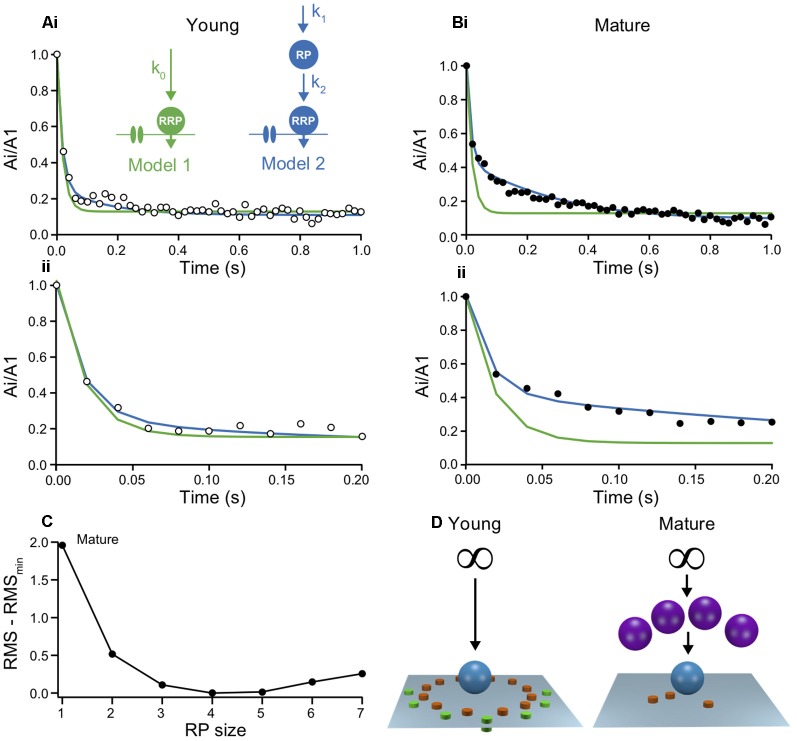
Models of short-term plasticity in young and mature synapses indicate a maturation of the reserve pool. **(A)** Two models were probed for their capability to describe the time-course of depression. **(i)** The models were fit to the normalized experimental Ai/A1 ratios from young L5PNs by simulating the processes at a single release site under the assumption that all sites are identical. Inset: schemes of model 1 (green) and model 2 (blue), showing Ca^2+^ channels, vesicles of the RRP and the reserve pool (RepP), that were replenished with Ca^2+^-independent rate constants. The infinite reserve pool is not shown. Fit parameters: k_0_ = 4.5 s^−1^ (green); RepP = 2 * RRP, k_1_ = 3.6 s^−1^, k_2_ = 4.6 s^−1^ (blue). **(ii)** Initial 200 ms shown on expanded time scale. **(B)** As in **(A)**, but for mature L5PNs. Note, that model 1 (green) overestimated the initial depression while model 2 (blue) reproduced the data reliably. Fit parameters: k_0_ = 4.5 s^−1^ (green); RepP = 4 * RRP, k_1_ = 0.9 s^−1^, k_2_ = 4.5 s^−1^ (blue). **(C)** Quality of fits with model 2 to the data from mature synapses with different RepP sizes per release site. Root-mean square deviations (RMS) between simulations and data were calculated for model fits with the indicated RepP sizes and the smallest RMS value (RMS_min_) was subtracted from these values. Note the clear minimum at an RepP size of 4. **(D)** Schemes illustrating the proposed maturation of the RepP at individual release sites during development from young (left) to mature (right; P/Q-type channels in orange, N-type channels in green, micro- and nanodomains; cf. Bornschein et al., 2019).

A process suitable to explain the biphasic time-course of EPSC depression in mature synapses could be that they can draw from a RepP of SVs intercalated between reserve pool and RRP or else that the RRP could be subdivided into slowly and rapidly releasing SVs (Neher and Brose, 2018; Schmidt, 2019). We tested this idea by fitting models of release and replenishment to the experimental data (Figure 4). The aim of these simulations was to identify a set of minimum requirements for obtaining the age-dependent differences in synaptic efficacy of L5PNs.

In the simplest model, emptied release sites in the RRP were replenished from an inexhaustible reserve pool of SVs *via* a basal, Ca^2+^-independent mechanism (model 1; Figure 4A). This simple model reproduced the time-course of depression and its steady-state reasonably well at young synapses. As would have been expected from fitting a model with a single rate constant to a biphasic decay, the drop to steady-state in the best model fit was somewhat faster than in the data. This discrepancy, however, was fairly negligible, indicating that the second component is circumstantial in young synapses (Figure 4Aii). Accordingly, however, the discrepancy between data and model 1 became substantial for mature synapses. Model 1 clearly failed to capture a large part of the time-course of depression (Figure 4B).

SVs were found to be organized more complex than being merely distributed between RRP and reserve pool (Rizzoli and Betz, 2005; Neher, 2015). Therefore, in model 2 we introduced a finite RepP between the infinite reserve pool and the RRP, similar to recent findings at cerebellar parallel-fiber synapses (Miki et al., 2016; Doussau et al., 2017). Both, the transition of SVs from reserve pool to emptied sites in the RepP and from the RepP to emptied sites in the RRP were assumed to be Ca^2+^ independent (Miki et al., 2016; Ritzau-Jost et al., 2018). Model 2 excellently fitted the data from mature synapses, with the best fit being obtained if the RepP was 4-fold larger than the RRP (Figure 4C). Under the assumption that the y-intercept represents the RRP, the RepP contains 56–184 SVs. At young synapses, model 2 appeared to also yield an improved description of the data (Figure 4Aii). However, the quality of the fit was almost independent of the size of the RepP. The lack of dependency on this parameter indicates that the system is overdetermined, which is consistent with the already good description of the data from young synapses by the simpler model 1. Thus, these simulations suggest that the RepP is required to describe transmission at mature synapses, while the replenishment pathway *via* RepP appears to be not yet established at young synapses (Figure 4D).

## Discussion

Our results suggest that the developmental increase in synaptic efficacy at excitatory neocortical synapses between L5PNs results from the functional maturation of a finite replenishing pool (RepP), intercalated between the reserve pool and the RRP. Whether the RepP and the RRP are different pools or constitute sub-pools of the same pool and whether their alignment is in series or in parallel is controversial at present (Neher, 2015; Neher and Brose, 2018; Schmidt, 2019). In the absence of experimental evidence for differences in other mechanisms of STP, including receptor desensitization and Ca^2+^-signaling (Figure 1), and the initial sizes of the RRP and its replenishment (Figure 2), our basic conclusion was drawn from analysis of the time-course of depression (Figure 3) and from fitting models with minimum requirements to the experimental data (Figure 4). Hence, we did not aim at excluding sub-pools or parallel arrangements, nor can we exclude a contribution of more sophisticated mechanisms like activity-dependent “a posteriori” modifications (Wölfel et al., 2007). However, our results hint towards the minimal requirements for sustained high-fidelity release from small cortical terminals operating with a small number of *N*_occ_.

On the presynaptic site, the STP characteristics of a synapse result from a convolute of *p*_v_, *N*_occ_ and the replenishment of *N*_occ_ or else the recruitment of new *N*_occ_. Traditionally, *p*_v_ was considered most important for setting the STP characteristics since replenishment rates were considered rather slow, hence, having only minor impact on high-frequency PPRs (e.g., Zucker and Regehr, 2002; Feldmeyer and Radnikow, 2009). However, recent results from small cerebellar cortical synapses revealed that their rate constants of replenishment/recruitment are much faster than initially thought, making replenishment the major determinant of STP with overfilling of the initial RRP on the millisecond time-scale *via* ultra-rapid recruitment of *N*_occ_ that increased *N* above its baseline value (Valera et al., 2012; Brachtendorf et al., 2015; Miki et al., 2016; Doussau et al., 2017). In our experiments we found no indications for alterations in *N* during paired pulses (Supplementary Figure S1). In this respect, L5PN synapses differ from parallel-fiber synapses (Valera et al., 2012; Brachtendorf et al., 2015) but behave similar to the synapse between the cerebellar cortical projection neurons, the Purkinje cells (Bornschein et al., 2013). Notably, the ultra-rapid replenishment/recruitment at parallel-fiber synapses occurs *via* a finite RepP (Miki et al., 2016; Doussau et al., 2017), very similar to our present findings at a neocortical synapse. Different from the parallel-fiber synapses, we assumed replenishment to be Ca^2+^-independent. Replenishment of SVs was found to be Ca^2+^-dependent at some CNS synapses (Sakaba, 2008; Miki et al., 2016; Doussau et al., 2017), while not at others (Ritzau-Jost et al., 2018). Hence, in the absence of experimental evidence for synapses of L5PNs, we kept the parameter space as simple as possible. In light of these results it appears that the size of the RepP and the magnitude of the replenishment rate are major determinants of STP of small cortical synapses.

To conclude, the presence of a RepP was described at cerebellar parallel-fiber synapses (Miki et al., 2016; Doussau et al., 2017) and a subdivision of the RRP into slow and fast pool at the brainstem calyx of Held (Sakaba, 2006; Wölfel et al., 2007) and cerebellar mossy fiber boutons (Hallermann et al., 2010; Ritzau-Jost et al., 2018). Our present data indicate a similar subdivision also for a neocortical synapse. Hence, a more complex organization of SVs into different sub-pools appears to be rather common at CNS synapses. Most notably, our data further suggest that the complex organization of SVs develops from simpler arrangements of SV pools during postnatal synapse maturation.

## Data Availability Statement

The raw data supporting the conclusions of this article will be made available by the authors, without undue reservation, to any qualified researcher.

## Ethics Statement

The animal study was reviewed and approved by University of Leipzig and State Directorate of Saxony, Germany; license T09/16.

## Author Contributions

HS: conceptualization, methodology, writing-review, editing and supervision. GB and SB: investigation, formal analysis and visualization. HS and GB: writing-original draft.

## Conflict of Interest

The authors declare that the research was conducted in the absence of any commercial or financial relationships that could be construed as a potential conflict of interest.

## Footnotes

^1^http://www.neuromatic.thinkrandom.com

## Supplementary Material

FIGURE S1 M-V data of first and second EPSC amplitudes fall to the same parabola. **(A)** Example M-V plot of first (black, A1) and second (red, A2) EPSC amplitudes derived from 20 ms ISI paired-pulse experiments at different [Ca^2+^]_e_ (1, 2 and 5 mM) from a young L5PN pair. **(B)** Same as in **(A)**, but for a mature L5PN pair in 0.5, 1, 2 and 5 mM [Ca^2+^]_e_. **(C)** Same as in **(B)**, but in the presence of 2 mM γDGG and for 1–10 m– [Ca^2+^]_e_. Note that in all plots the data of the first and second EPSC amplitudes fall to the same parabola (see Bornschein et al., 2019).

FIGURE S2 Analysis of spontaneous EPSCs (sEPSCs) after high-frequency trains. **(A)** Example traces with evoked (arrowheads) and sEPSCs recorded from a young L5PN pair at the end of a high-frequency train. The first two arrowheads mark the timepoints of the last two presynaptic APs of the preceding high-frequency train. Recordings were continued for another 1.4 s during which two APs (arrowheads) were evoked in the presynaptic cell at increased intervals. **(Bi)** Analysis of sEPSC amplitudes from the cell pair shown in **(A)**. **(ii)** Same as in **Bi** but for a mature cell pair. **(C)** Summary of correlation analysis between sEPSC amplitudes and time of young (*n* = 4) and mature cell pairs (*n* = 5), showing Pearson’s correlation coefficient (Pr) and *P*-values (P).

TABLE S1 Multi-Pulse ratios (Ai/A1) during HF stimulation.

TABLE S2 Synaptic failures (F_syn_) during HF stimulation.
