# Supplementary figures and images for: Developmental Increase of Neocortical Presynaptic Efficacy via Maturation of Vesicle Replenishment
Source: Front Synaptic Neurosci. 2020 Jan 15;11:36. doi: 10.3389/fnsyn.2019.00036 (PMC6974464; doi:10.3389/fnsyn.2019.00036)

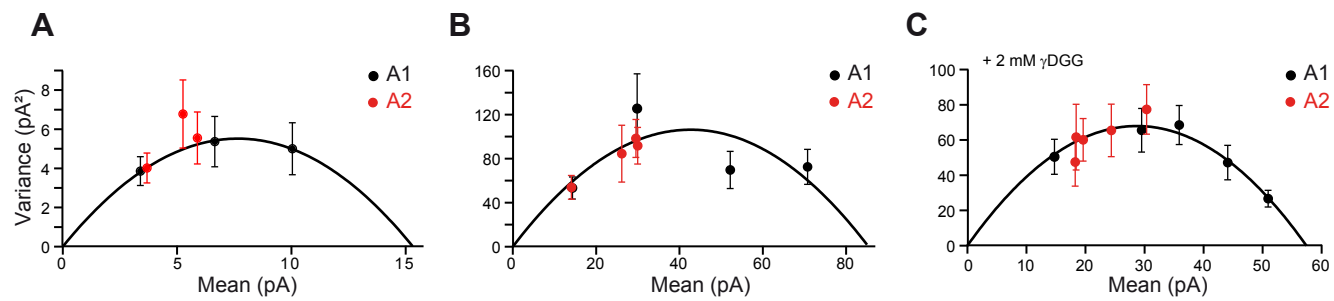

**Figure S1**

Supplement: FIGURE S1 — M-V data of first and second EPSC amplitudes fall to the same parabola. (A) Example M-V plot of first (black, A1) and second (red, A2) EPSC amplitudes derived from 20 ms ISI paired-pulse experiments at different [Ca2+]e (1, 2 and 5 mM) from a young L5PN pair. (B) Same as in (A), but for a mature L5PN pair in 0.5, 1, 2 and 5 mM [Ca2+]e. (C) Same as in (B), but in the presence of 2 mM γDGG and for 1–10 m– [Ca2+]e. Note that in all plots the data of the first and second EPSC amplitudes fall to the same parabola (see Bornschein et al., 2019). [file Data_Sheet_1.PDF]

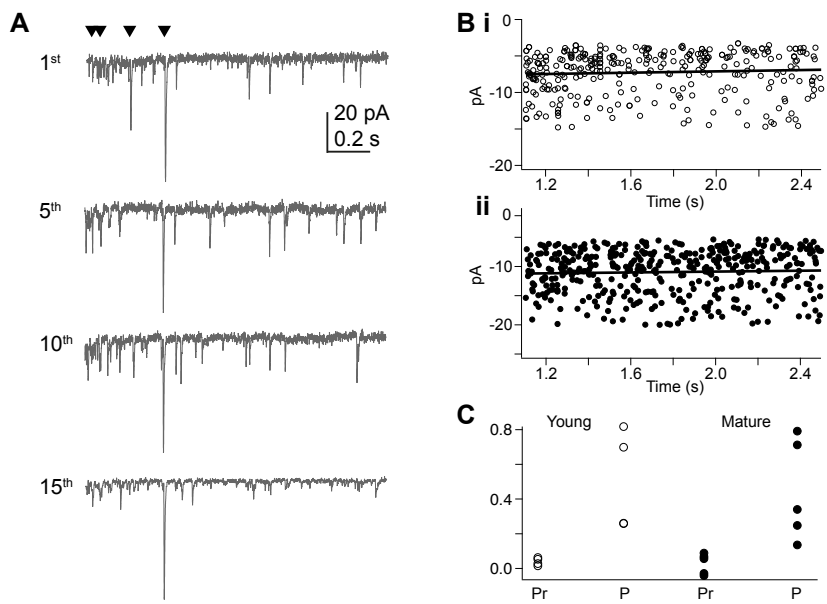

**Figure S2**

Supplement: FIGURE S2 — Analysis of spontaneous EPSCs (sEPSCs) after high-frequency trains. (A) Example traces with evoked (arrowheads) and sEPSCs recorded from a young L5PN pair at the end of a high-frequency train. The first two arrowheads mark the timepoints of the last two presynaptic APs of the preceding high-frequency train. Recordings were continued for another 1.4 s during which two APs (arrowheads) were evoked in the presynaptic cell at increased intervals. (Bi) Analysis of sEPSC amplitudes from the cell pair shown in (A). (ii) Same as in Bi but for a mature cell pair. (C) Summary of correlation analysis between sEPSC amplitudes and time of young (n = 4) and mature cell pairs (n = 5), showing Pearson’s correlation coefficient (Pr) and P-values (P). [file Data_Sheet_2.PDF]
