# Supplementary material for: Developmental Increase of Neocortical Presynaptic Efficacy via Maturation of Vesicle Replenishment
Source: Front Synaptic Neurosci. 2020 Jan 15;11:36. doi: 10.3389/fnsyn.2019.00036 (PMC6974464; doi:10.3389/fnsyn.2019.00036)
Supplement: TABLE S1 — Multi-Pulse ratios (Ai/A1) during HF stimulation. [file Table_1.DOCX]

**Table S1. Multi-Pulse ratios (Ai/A1) during HF stimulation.**

| **Group** | | **N** | **Median** | **IQR 25%** | **IQR 75%** | **P** (MWU  vs. young) |
| --- | --- | --- | --- | --- | --- | --- |
| A2/A1 | Young | 14 | 0.415 | 0.329 | 0.509 |  |
|  | Mature | 7 | 0.538 | 0.505 | 0.701 | 0.012 |
| A3/A1 | Young | 14 | 0.296 | 0.236 | 0.366 |  |
|  | Mature | 7 | 0.455 | 0.392 | 0.459 | <0.001 |
| A4/A1 | Young | 14 | 0.184 | 0.113 | 0.284 |  |
|  | Mature | 7 | 0.423 | 0.328 | 0.476 | <0.001 |
| A5/A1 | Young | 13 | 0.175 | 0.124 | 0.276 |  |
|  | Mature | 3 | 0.341 | 0.259 | 0.473 | 0.009 |
| A6/A1 | Young | 14 | 0.158 | 0.127 | 0.277 |  |
|  | Mature | 7 | 0.317 | 0.260 | 0.399 | 0.028 |
| A7/A1 | Young | 14 | 0.207 | 0.074 | 0.268 |  |
|  | Mature | 7 | 0.309 | 0.279 | 0.337 | 0.015 |
| A8/A1 | Young | 14 | 0.166 | 0.120 | 0.245 |  |
|  | Mature | 7 | 0.247 | 0.208 | 0.335 | 0.068 |
| A9/A1 | Young | 14 | 0.187 | 0.091 | 0.291 |  |
|  | Mature | 7 | 0.257 | 0.237 | 0.283 | 0.168 |
| A10/A1 | Young | 14 | 0.192 | 0.125 | 0.253 |  |
|  | Mature | 7 | 0.250 | 0.215 | 0.294 | 0.093 |
| A50/A1 | Young | 14 | 0.127 | 0.089 | 0.189 |  |
|  | Mature | 7 | 0.129 | 0.097 | 0.163 | 0.970 |

**Table S2. Synaptic failures (F_syn_) during HF stimulation.**

| **Group** | | **N** | **Median** | **IQR 25%** | **IQR 75%** | **P** (MWU  vs. young) |
| --- | --- | --- | --- | --- | --- | --- |
| F_syn_2 | Young | 13 | 0.083 | 0.000 | 0.236 |  |
|  | Mature | 8 | 0.000 | 0.000 | 0.000 | 0.051 |
| F_syn_3 | Young | 13 | 0.167 | 0.033 | 0.354 |  |
|  | Mature | 8 | 0.000 | 0.000 | 0.115 | 0.026 |
| F_syn_4 | Young | 13 | 0.300 | 0.100 | 0.408 |  |
|  | Mature | 8 | 0.000 | 0.000 | 0.072 | 0.001 |
| F_syn_5 | Young | 13 | 0.300 | 0.158 | 0.431 |  |
|  | Mature | 8 | 0.029 | 0.000 | 0.135 | 0.007 |
| F_syn_6 | Young | 13 | 0.300 | 0.225 | 0.427 |  |
|  | Mature | 8 | 0.000 | 0.000 | 0.044 | <0.001 |
| F_syn_7 | Young | 13 | 0.333 | 0.191 | 0.550 |  |
|  | Mature | 8 | 0.000 | 0.000 | 0.107 | <0.001 |
| F_syn_8 | Young | 13 | 0.364 | 0.150 | 0.569 |  |
|  | Mature | 8 | 0.039 | 0.000 | 0.089 | <0.001 |
| F_syn_9 | Young | 13 | 0.300 | 0.150 | 0.388 |  |
|  | Mature | 8 | 0.039 | 0.000 | 0.166 | 0.011 |
| F_syn_10 | Young | 13 | 0.364 | 0.100 | 0.456 |  |
|  | Mature | 8 | 0.029 | 0.000 | 0.156 | 0.002 |
| F_syn_11 | Young | 13 | 0.308 | 0.150 | 0.618 |  |
|  | Mature | 8 | 0.042 | 0.000 | 0.109 | 0.004 |
| F_syn_12 | Young | 13 | 0.333 | 0.241 | 0.483 |  |
|  | Mature | 8 | 0.000 | 0.000 | 0.171 | 0.001 |
| F_syn_13 | Young | 13 | 0.400 | 0.348 | 0.400 |  |
|  | Mature | 8 | 0.039 | 0.000 | 0.163 | <0.001 |
| F_syn_14 | Young | 13 | 0.333 | 0.233 | 0.497 |  |
|  | Mature | 8 | 0.000 | 0.000 | 0.202 | 0.001 |
| F_syn_15 | Young | 13 | 0.385 | 0.200 | 0.519 |  |
|  | Mature | 8 | 0.039 | 0.000 | 0.208 | 0.004 |
| F_syn_16 | Young | 13 | 0.400 | 0.286 | 0.600 |  |
|  | Mature | 8 | 0.068 | 0.000 | 0.150 | <0.001 |
| F_syn_17 | Young | 13 | 0.400 | 0.200 | 0.533 |  |
|  | Mature | 8 | 0.039 | 0.000 | 0.208 | 0.005 |
| F_syn_18 | Young | 13 | 0.455 | 0.200 | 0.608 |  |
|  | Mature | 8 | 0.039 | 0.000 | 0.113 | 0.001 |
| F_syn_19 | Young | 13 | 0.400 | 0.200 | 0.550 |  |
|  | Mature | 8 | 0.080 | 0.015 | 0.150 | 0.003 |
| F_syn_20 | Young | 13 | 0.400 | 0.300 | 0.545 |  |
|  | Mature | 8 | 0.119 | 0.000 | 0.322 | 0.007 |
| F_syn_50 | Young | 13 | 0.545 | 0.427 | 0.700 |  |
|  | Mature | 8 | 0.360 | 0.200 | 0.490 | 0.029 |
